# Diagnosis of *Schistosoma* infection in non-human animal hosts: A systematic review and meta-analysis

**DOI:** 10.1371/journal.pntd.0010389

**Published:** 2022-05-06

**Authors:** Song Liang, Keerati Ponpetch, Yi-Biao Zhou, Jiagang Guo, Berhanu Erko, J. Russell Stothard, M. Hassan Murad, Xiao-Nong Zhou, Fadjar Satrija, Joanne P. Webster, Justin V. Remais, Jürg Utzinger, Amadou Garba

**Affiliations:** 1 Department of Environmental and Global Health, College of Public Health and Health Professions, University of Florida, Gainesville, Florida, United States of America; 2 Emerging Pathogens Institute, University of Florida, Gainesville, Florida, United States of America; 3 Sirindhorn College of Public Health Trang, Faculty of Public Health and Allied Health Sciences, Praboromarajchanok Institute, Trang, Thailand; 4 School of Public Health, Fudan University, Shanghai, People’s Republic of China; 5 Department of Control of Neglected Tropical Diseases, World Health Organization, Geneva, Switzerland; 6 Aklilu Lemma Institute of Pathobiology, Addis Ababa University, Addis Ababa, Ethiopia; 7 Department of Tropical Disease Biology, Liverpool School of Tropical Medicine, Merseyside, United Kingdom; 8 Division of Public Health, Infectious Diseases and Occupational Medicine, Mayo Clinic, Rochester, Minnesota, United States of America; 9 National Institute of Parasitic Diseases, Chinese Center for Disease Control and Prevention, Shanghai, People’s Republic of China; 10 Department of Animal Infectious Diseases and Veterinary Public Health, Faculty of Veterinary Medicine, IPB University, Bogor, Indonesia; 11 Department of Pathobiology and Population Sciences, Royal Veterinary College, Hertfordshire, United Kingdom; 12 School of Public Health, University of California, Berkeley, Berkeley, California, United States of America; 13 Swiss Tropical and Public Health Institute, Allschwil, Switzerland; 14 University of Basel, Basel, Switzerland; University of Iowa, UNITED STATES

## Abstract

**Background:**

Reliable and field-applicable diagnosis of schistosome infections in non-human animals is important for surveillance, control, and verification of interruption of human schistosomiasis transmission. This study aimed to summarize uses of available diagnostic techniques through a systematic review and meta-analysis.

**Methodology and principal findings:**

We systematically searched the literature and reports comparing two or more diagnostic tests in non-human animals for schistosome infection. Out of 4,909 articles and reports screened, 19 met our inclusion criteria, four of which were considered in the meta-analysis. A total of 14 techniques (parasitologic, immunologic, and molecular) and nine types of non-human animals were involved in the studies. Notably, four studies compared parasitologic tests (miracidium hatching test (MHT), Kato-Katz (KK), the Danish Bilharziasis Laboratory technique (DBL), and formalin-ethyl acetate sedimentation-digestion (FEA-SD)) with quantitative polymerase chain reaction (qPCR), and sensitivity estimates (using qPCR as the reference) were extracted and included in the meta-analyses, showing significant heterogeneity across studies and animal hosts. The pooled estimate of sensitivity was 0.21 (95% confidence interval (CI): 0.03–0.48) with FEA-SD showing highest sensitivity (0.89, 95% CI: 0.65–1.00).

**Conclusions/significance:**

Our findings suggest that the parasitologic technique FEA-SD and the molecular technique qPCR are the most promising techniques for schistosome diagnosis in non-human animal hosts. Future studies are needed for validation and standardization of the techniques for real-world field applications.

## Author summary

The diagnosis of schistosome infection in non-human hosts is important for control and elimination of human schistosomiasis. This study aimed to summarize effectiveness of available diagnostic techniques through a systematic review of the literature and reports comparing two or more diagnostic tests in non-human animals. Overall, 4,909 articles and reports were screened. Nineteen articles met our inclusion criteria and were analyzed in greater detail. A total of 14 techniques (parasitologic, immunologic, and molecular) and nine types of non-human animals were involved in the studies. Four studies comparing parasitologic tests (miracidium hatching test (MHT), Kato-Katz (KK), the Danish Bilharziasis Laboratory technique (DBL), and formalin-ethyl acetate sedimentation-digestion (FEA-SD)) with quantitative polymerase chain reaction (qPCR) were included in the meta-analyses. The findings suggest that the parasitologic technique FEA-SD and molecular techniques, especially qPCR, are the most promising techniques. However, further validation and standardization of the techniques under field conditions are needed.

## Introduction

Effective disease surveillance is a critical component of health systems, by providing timely information to monitor disease trends, guide interventions, evaluate health outcomes, and set disease control and elimination goals. The transmission of several parasitic diseases involves multiple species of hosts, in addition to humans. Hence, identifying infections in human and non-human hosts through accurate diagnostic tools is central to their surveillance. Schistosomiasis, caused by infection of blood flukes of the genus *Schistosoma*, is one such disease and poses health threats to an estimated 800 million people in the tropics and subtropics with more than 250 million people currently infected globally [1–3]. There are six major species that infect humans, among which, *Schistosoma mansoni*, *S*. *haematobium*, and *S*. *japonicum* have a wide geographic distribution, are of primary public health concerns, and account for the majority of human disease burdens [4–6]. Furthermore, it is becoming increasingly apparent that humans can be infected, often at very high prevalence levels, with viable hybrids between *S*. *haematobium* with the livestock schistosome species of *S*. *bovis*, *S*. *curassoni*, and *S*. *mattheii* across parts of sub-Saharan Africa, as well as rarer cases of human infections with viable hybridized *S*. *bovis* with *S*. *currassoni* [7–12].

With extensive global efforts to control schistosomiasis over the past 15–20 years, the world has witnessed a significant change in patterns of infection, including remarkable reductions in disease-associated mortality and morbidity that have been achieved in many endemic areas [13–16]. Some successful control programs, including areas that have achieved local elimination of transmission, have been observed in, for example, certain Caribbean island nations and Brazil in the Americas; Egypt, Morocco, Tunisia, Algeria, and Mauritius in North Africa and the Middle East; Saudi Arabia and Iran in the Persian Gulf; and Japan and the People’s Republic of China in Asia. Such success has encouraged the development of an agenda of schistosomiasis elimination at the global scale [13–15,17]. Yet, it has been well recognized that, given the complexity of factors involved in the *Schistosoma* transmission, disease elimination will require integrated efforts targeting multiple components and transmission processes [18–21]. A central element of control and elimination efforts is the identification of infection sources for intervention (e.g., finding infected individuals for treatment) through diagnosis of infections. However, as transmission levels are reduced with control efforts, and traditional techniques for diagnosis of schistosome infections butt up against their limits of detection, there is a pressing need for sensitive and field-applicable diagnostic procedures for humans [13,22–24] and possible animal reservoirs of the parasite [25–27]. Such tools would enable effective monitoring and surveillance, as well as verification of elimination of the disease transmission (e.g., no circulation of the pathogen).

Natural infections of non-human animals by the three species of *Schistosoma* parasites (*S*. *mansoni*, *S*. *haematobium*, and *S*. *japonicum*) have been reported with varying public health implications based on available evidence. *S*. *japonicum* is the causative agent of schistosomiasis in Asia, primarily in the People’s Republic of China, the Philippines, and parts of Indonesia [18,28,29], and is widely recognized as a zoonotic parasite [4,27,30]. Over 40 species of wild and domestic mammalian animals can serve as reservoirs of *S*. *japonicum* [26,30,31]. Studies in the People’s Republic of China [20,21,32–35] and the Philippines [36–39] have revealed that some mammalian reservoirs, in particular water buffaloes, have played important roles in the local transmission of schistosomiasis to humans. For example, studies have suggested that bovines may play an important role in sustaining transmission in the lower Yangtze River region of the People’s Republic of China [40–43], contributing to from around 60% of infection sources in the Poyang Lake area [33,34] to 90% in Anhui province [44]. Other mammalian hosts, such as horses, pigs, dogs, cats [35,44], and wild rodents [40–43,45], have been observed with varying levels of infections from field surveys, and their contributions to human infections have received less interest to date compared to bovine hosts.

In hilly and mountainous regions in the southwestern part of the People’s Republic of China, longitudinal surveillance data have shown a high correlation between prevalence of infection in humans and bovines and rodents at the regional scale [43,46], and studies have suggested that bovines and rodents were a key factor underlying re-emergence of schistosomiasis transmission in areas previously controlled or locally eliminated [32,47]. Field studies in the Philippines have also reported a wide range of *S*. *japonicum-*infected animal species, with buffaloes and dogs both likely important players in parasite transmission to humans [28,39,48–50].

Natural infections of *S*. *mansoni*, *S*. *haematobium*, and also notably *S*. *haematobium* with *S*. *bovis* hybrids, have been observed in some non-human animals such as primates (e.g., baboons), rodents, and pigs [51–57]. Further, there is also evidence that human *S*. *mansoni* is maintained in non-human primates, e.g., in East Africa [57], that shared phylogenetic genotypes are matched, indicative of shared transmission between humans and rodents in West Africa [58]. Yet, the contribution of these animals to the epidemiology of human schistosomiasis transmission remains poorly understood, and further research is needed to estimate the burden of disease attributable to non-human animal circulation [25,52,59]. Accurate detection of *Schistosoma* infection, to the species and ideally strain/genotype level, in animals would provide critical information to guide surveillance and inform control and elimination [7,27,46,58,60].

Diagnosis of *Schistosoma* infection in humans and diagnosis of animal infections rely on techniques that fall in three categories: parasitologic, immunologic, and molecular. Parasitologic techniques typically involve microscopy such as the Kato-Katz (KK) thick smear test and miracidium hatching test (MHT); immunodiagnostic techniques detect species-specific antigens or antibodies; and molecular techniques use parasite DNA for detection. These techniques have been widely used in field settings either separately or in combination, exhibiting varying levels of effectiveness and utility. Similar challenges as those facing the diagnosis in humans arise when seeking to diagnose infections of animal hosts (e.g., insensitivity of the KK and MHT with decreasing infection intensities). Furthermore, diagnosis of infection in animal hosts presents additional challenges, typically associated with sample collection and processing. To assess the effectiveness of currently available techniques for *Schistosoma* diagnosis of non-human animals, we pursued a systematic review and meta-analysis of the literature on diagnosis of animal *Schistosoma* infections.

## Methods

### Search strategy and selection criteria

A systematic literature review was pursued with the aim to identify relevant studies, spanning from January 1990 to December 2020, that examined *Schistosoma* infections in non-human animal hosts using diagnostic techniques and assess their relative effectiveness in the diagnosis. The PRISMA guidelines [61] for systematic reviews were followed to report this review. We performed searches in the following three electronic databases: PubMed, Web of Science, and Science Direct. Embase and Cochrane databases were also used in the initial search, but did not yield extra coverage of studies beyond the aforementioned databases. We also searched the electronic archives of relevant international agencies, including the World Health Organization (WHO)’s Library Database, the Food and Agriculture Organization (FAO), and World Organization for Animal Health (OIE). Considering that the People’s Republic of China is a major *S*. *japonicum* endemic country and many relevant studies were published in Chinese, we searched China National Knowledge Infrastructure (CNKI) and Wanfang for Chinese language papers. Books, dissertation, and conference abstracts were also considered.

The following keywords and combinations were used in the search: “schistosomiasis”, “schistosome”, “*Schistosoma*”, in combination with “diagnosis”, “detection”, “infection”, “veterinary screening”, “parasitologic assay”, “immunoassay”, “molecular assay”, and “non-human animal”, “animal reservoir(s)”, and “animal host(s)”. Searches included appropriate wildcards and truncations, and the bibliographies of identified documents were hand-searched for additional references. No language restriction was imposed for database searches. Titles of papers retrieved from each database were manually screened first to remove irrelevant references. Then, abstracts were further screened and the full texts of potentially relevant papers were reviewed. This process was conducted independently by two reviewers (KP and YBZ).

**Fig 1:**
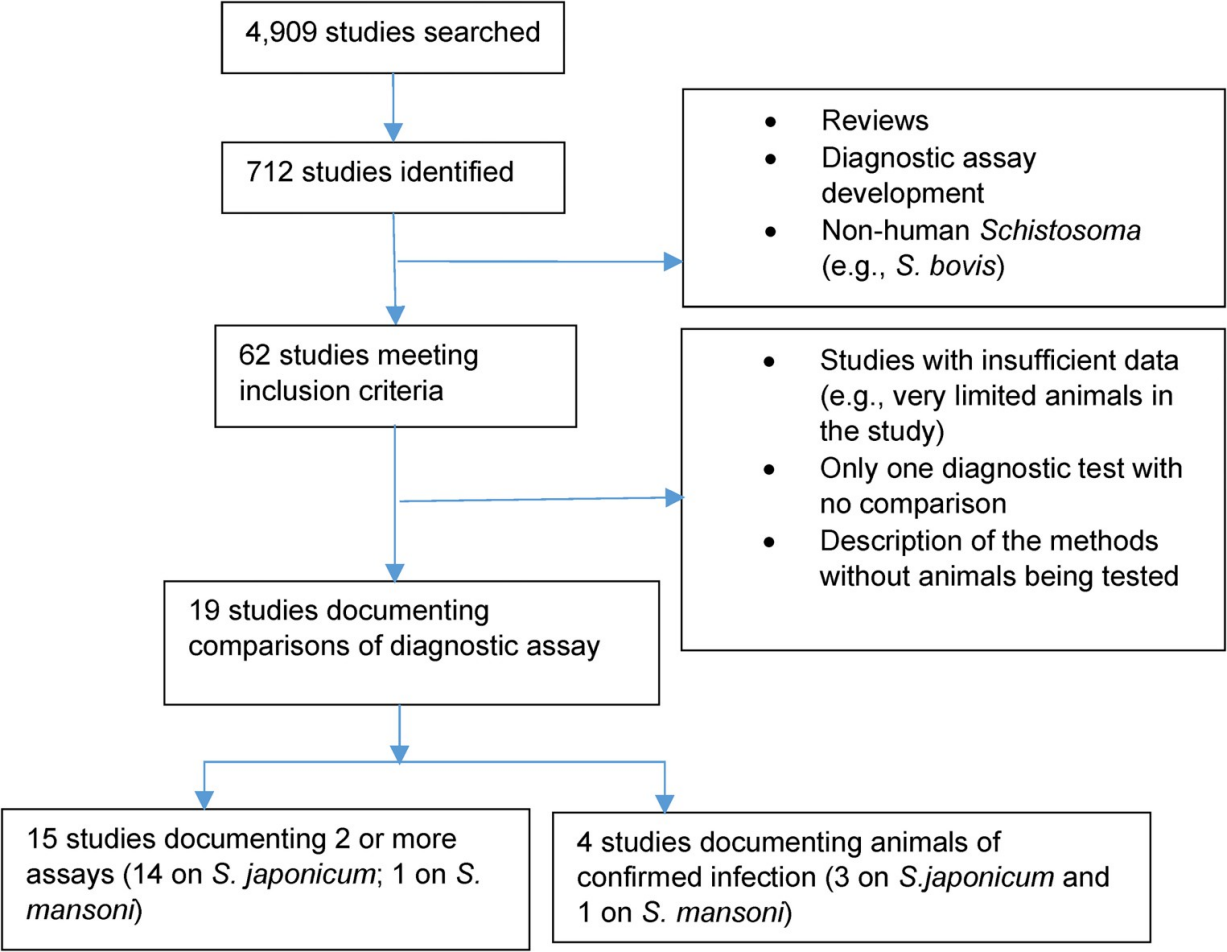
Inclusion and exclusion of studies on diagnosis of *Schistosoma* infections in animals and search results.

The inclusion and exclusion criteria of articles/studies in the present review are summarized in Fig 1. We considered field-based epidemiologic studies (e.g., cross-sectional) and laboratory-based studies involving diagnosis of *Schistosoma* infection in non-human animals, as well as relevant veterinary screening (e.g., non-research) and veterinary medicine research. As we were interested in relative effectiveness of diagnostic techniques, only publications, if in the absence of a diagnostic ‘gold’ standard of infection, reporting the use of at least two diagnostic tools (e.g., parasitologic, or immunologic, or molecular (e.g., PCR-based) tests, or a combination of them) in the same study were included. Publication reporting, if with confirmed animal infections (as the ‘gold’ standard, e.g., confirmed artificial infection), two or more diagnostic tests were eligible for inclusion. The study search and selection were performed by two independent reviewers.

### Data extraction and types of outcome measures

Following the identification of eligible studies, relevant information was extracted, including year of publication, study area (e.g., site and country), study design (e.g., field- or laboratory-based), diagnostic tests, animal species, and sample size (e.g., number of specific animal host examined), and outcome measured (e.g., numbers of positive and negative tests), and entered into a standardized Excel spreadsheet by authors, independently. The primary outcome was proportion of test positive (test positive/examined x 100%) by diagnosis through a specific test (e.g., parasitologic, immunologic, or molecular). Studies with comparable information (involving consistent comparisons across diagnostic techniques, e.g., a study comparing a few parasitologic techniques with a molecular technique, qPCR) were included in the assessment of agreement test and relative sensitivity of diagnosis, cross-tabulated information was extracted.

### Statistical analysis

Statistical analyses were limited to a subset of studies with comparable tests. The comparisons included parasitologic assays vs. immunoassays (limited to MHT vs. enzyme-linked immunosorbent assay (ELISA), the colloidal gold immunochromatography assay (GICA), the dot immunogold filtration assay (DIGFA), and dye dipstick immunoassay (DDIA)), and molecular assays vs. parasitologic assays (limited to qPCR vs. MHT, KK, the Danish Bilharziasis Laboratory test (DBL), and formalin-ethyl acetate sedimentation-technique(FEA-SD), see Table 1 for details). Cohen’s Kappa estimate was used to assess the degree of agreement between two tests across the studies with comparable information [62]. For studies with comparable data on the tests, which were limited to four parasitologic techniques (MHT, KK, DBL, and FEA-SD), sensitivity was estimated using qPCR result as the reference given the availability of comparable data and qPCR being reported to be a highly sensitive technique in the detection of schistosome infection in animals [63–66]. The Kappa and sensitivity summary statistics across comparisons were estimated using generic inverse variance weighting method. Heterogeneity across comparisons was tested using *I*^*2*^ statistic (e.g., inconsistency or variability in effect estimates across studies).

**Table 1:** Key characteristics of identified studies on diagnosis of schistosome infections in non-human animals.

Species	Location	Study	Host	Sample size	Parasitologic assay	Immunoassay	Molecular assay	Ref
***S*.*j*.**	Dongting Lake, China	Field	Rodent Dog Goat Buffalo Cattle	76 52 145 10 10	KK MHT		qPCR PCR	[45]
***S*.*j*.**	Hunan, China	Exp, Field	Mouse Rabbit Buffalo Goat	70 50 132 117		ELISA GICA		[73]
***S*.*j*.**	Leyte, Philippines	Field	Cattle Buffalo	48 105	FEA-SD		qPCR	[72]
***S*.*j*.**	Samar, Philippines	Field	Buffalo	44	KK MHT		PCR qPCR	[50]
***S*.*j*.**	Sichuan, China	Field	Buffalo	8	MHT		PCR	[63]
***S*.*j*.**	Cagayan, Philippines	Field	Buffalo	50		ELISA COPT	PCR	[74]
***S*.*m*.**	Lake Victoria, Uganda	Field	Chimpanzee	39	KK MHT	ELISA	qPCR	[75]
***S*.*m*.**	Brazil	Exp	Rodent	23	KK	ELISA	PCR	[76]
***S*.*j*.**	Hubei, China	Field	Buffalo	178		ELISA	PCR	[77]
***S*.*j*.**	Leyte, Philippines	Field	Buffalo	81	KK MHT DBL		qPCR	[66]
***S*.*j*.**	Hunan, China	Field	Goat Buffalo Cattle	314 197 162	MHT	GICA IHA		[70]
***S*.*j*.**	Zhejiang, China	Exp	Cattle	110		ELISA DIGFA		[78]
***S*.*j*.**	China	Exp	Sheep	107		ELISA GICA		[79]
***S*.*j*.**	Zhejiang, China	Field	Cattle	139	MHT	ELISA T-DIGFA		[69]
***S*.*j*.**	Hunan, China	Field	Cattle	110	MHT	ELISA		[68]
***S*.*j*.**	Hunan, China	Field	Cattle Pig	33 50	MHT	DDIA		[80]
***S*.*j*.**	Jiangxi, China	Field	Cattle	2,277		IHA PAPS		[81]
***S*.*j*.**	Zhejiang, China	Field	Cattle Rabbit	94 96		ELISA IHA		[82]
***S*.*j*.**	Hubei, China	Field	Cattle	4,217	MHT	PAPS		[71]

**Species**, *S*.*j*.*–Schistosoma japonicum*; *S*.*m*.*–S*. *mansoni*

**Study**, field–field-based epidemiologic study (primarily cross-sectional studies); Exp–experiment-based studies; cases (animals experimentally infected) and controls (not infected) were included

**Parasitologic assay**, KK–Kato-Katz technique; MHT–miracidium hatching test; DBL–Danish Bilharziasis Laboratory technique; FEA-SD–formalin-ethyl acetate sedimentation technique.

**Immunoassay**, ELISA–enzyme-linked immunosorbent assay; GICA–gold immunochromatography assay; COPT–circumoval precipitin test; DDIA–dipstick dye immunoassay; IHA–indirect hemagglutination; PAPS–polyacetal polystyrene immunization microspheres; DIGFA–dot immunogold filtration assay; CCA–circulating cathodic antigen

**Molecular assay**, PCR–polymerase chain reaction; qPCR–real-time (or quantitative) polymerase chain reaction

Pooled estimates of sensitivity of selected diagnostic tests were analyzed in a meta-analysis using a random-effects model. Due to heterogeneity of diagnostic tests across the included studies and limited studies with comparable information, we restricted our analysis to the studies with comparable information for the meta-analysis. The meta-analysis was performed in R [67].

## Results

### Search results

The search process and results are shown in Fig 1. The search terms returned 4,909 records from all databases, reducing to 712 records after title screening and removal of duplicates. Through further examination by removing review-type articles, articles pertaining to development of diagnostic assays (e.g., not for field and/or laboratory applications) and non-human *Schistosoma* species, 62 studies were identified for full-text review. Of these, 43 articles contained insufficient information or only one diagnostic test, and hence, were excluded from our review. The remaining 19 studies were included for qualitative analysis in this review and four of them were included in the meta-analysis [45,50,66,68–72]. Note that, for the four studies with confirmed infections prior to diagnostic testing (on lower right box, Fig 1), more than two diagnostic techniques were also examined in each study.

### General characteristics

This systematic review identified 19 studies published between 1993 and 2020 with key characteristics of the included studies summarized in Table 1. Among the included studies, 15 were field-based (e.g., cross-sectional surveys of non-human animals), three were based on experimentally infected animals, and one involved both. Seventeen of them were based on *S*. *japonicum*, involving a range of hosts, including domestic animals (e.g., buffalo, cattle, dog, goat, and pig), laboratory animals (e.g., mouse and rabbit), and wild animals (e.g., rodent) in the People’s Republic of China and the Philippines. The remaining two papers examined *S*. *mansoni* in chimpanzee and rodents in Uganda and Brazil, respectively (Table 1).

All studies involved the use of two or more diagnostic techniques in the three categories of diagnostic techniques: (i) parasitologic (n = 4); (ii) immunologic (n = 8); and (iii) molecular (n = 2). Key characteristics of these techniques are summarized in Box 1. Among the 19 studies, two studies used all three types of techniques, five studies used both parasitologic and molecular techniques, five used both parasitologic and immunologic techniques, two studies used both immunologic and molecular techniques, and five studies used two immunologic techniques (Table 1). Sample sizes varied substantially across the studies and different animal hosts, with the majority of animals being buffalo and cattle, accounting for 87.7% (8,145/9,284) of the total number of animals examined. Thirty-nine non-human primate (all chimpanzees) were examined for *S*. *mansoni* in Uganda [75].

**Box 1: Key characteristics of diagnostic techniques in the included studies**

As diagnosis of *Schistosoma* infection in humans, development and utilization of diagnostic tools for *Schistosoma* infections in non-human animals are also important for surveillance and control of the disease involving zoonotic transmission. The development and use of diagnostic tools for animal infections have, to some extent, paralleled those of the diagnosis of human *Schistosoma* infections. Many techniques have been developed and/or adapted (e.g., from diagnostic techniques of human infection) and these techniques can be broadly grouped into three major categories–parasitologic, immunologic, and molecular techniques. The techniques used in the studies included in this review are summarized below.

Parasitologic techniques

As for the diagnosis of human schistosome infection, detection of excreted schistosome eggs from fecal samples of animal hosts is a customary method, which is direct and specific. Four techniques were used in the 13 studies–the KK thick smear test, the MHT, the DBL technique, and the FEA-SD technique.

The Kato-Katz technique (KK). The KK is the standard method recommended by WHO for both qualitative and quantitative diagnosis of intestinal schistosomiasis (e.g., *S*. *japonicum* and *S*. *mansoni*) and the most widely used method in the field for diagnosis of human infections with these species [23,83]. The KK was also adapted for detection of schistosome infection in non-human animal hosts. Briefly, fresh, homogenized stool sample is pressed through approximately 60–105 μm mesh and filled in a standard 41.7 mg volume template or other volume templates designed to contain a specific amount of stool on a microscopic slide. The resulting sample on the slide is then covered with glycerin/methylene blue-soaked cellophane (to clear the fecal samples) and pressed to spread the stool evenly on the slide, which is then examined under a microscope. One or multiple slides for each stool sample are prepared and systematically screened by experienced microscopists with the egg count expressed typically as the number of eggs per gram of stool (EPG) through multiplication (e.g., 24 for a single KK in the case of using 41.7 mg volume template).

Miracidium hatching test (MHT). The MHT is another commonly used method for detection of schistosome (in particular *S*. *japonicum*) infections in humans and animals. The technique checks for live miracidia through egg hatching to detect infection. Briefly, homogenized stool samples, usually ~50 g, are sieved through 1–2 layers (with slight variations over different studies/applications) of mesh, the sediment is then collected and placed to an Erlenmeyer flask filled with fresh, unchlorinated water (with pH around 7.0), and is subjected to artificial or natural light at room temperature controlled in the range of 25°C to 30°C. The neck of the flask is then examined at intervals (typically 1, 4, 8, and 12 hours) to detect the presence of miracidia [84]. Slight modifications, depending on field and laboratory logistics, have been made across field applications [26,32,35,45,50].

The Danish Bilharziasis Laboratory (DBL) technique. The DBL technique was initially developed for the evaluation of *S*. *japonicum* eggs in pig feces. The basic procedure involves filtration, sedimentation, and centrifugation [85]. Briefly, 5 g of feces taken from homogenized specimens is mixed with 100 ml of 1.2% saline, agitated for 5–10 min, and gone through 3 layers of sieves at 400 μm, 100 μm, and 45 μm mesh size, respectively. The residue materials remaining on the 45 μm mesh sieve is then removed to a sedimentation flask, filled with saline to allow sedimentation in the dark. The sediment is then centrifuged and re-suspended to obtain a final volume of 2.25 ml. After thoroughly mixing, 150 μl of the solution is removed to a 1 ml microscope chamber slide mixed with 850 μl of saline. Three chamber slides are examined under a microscope and the number of schistome eggs are counted to obtain an intensity measure, the total number of EPG [36,85].

Formalin-ethyl acetate sedimentation-digestion (FEA-SD) technique. A procedure has been developed for quantification of *S*. *japonicum* eggs from bovine feces. The basic procedure involves filtration, sedimentation, potassium hydroxide digestion, centrifugation, and then microscopy [86]. Briefly, ~50 g of homogenized fecal sample is sieved through 40–60 copper mesh (234–380 μm) sieve and subsequently a 260 copper mesh (61 μm) sieve. The sediment on the second layer mesh is then washed into a conical flask, mixed through shaking, and sedimented for 30 min. The sediment is re-suspended in 10% (v/v) formalin and natural sedimentation repeated twice. The resulting suspension is subjected to two rounds of centrifugations and digestion using potassium hydroxide, and further centrifugation before microscopy. The detailed operation procedures are reported by Xu et al. [86].

Immunologic techniques

Like diagnosis of human schistosome infections, a range of immunodiagnostic techniques have been developed, targeted on anti-schistosome antibodies or schistosome antigens present in serum/urine for infection detection of non-human animals. In this review, eight immunodiagnostic techniques were reported in the included studies; namely (i) ELISA; (ii) circumoval precipitin test (COPT); (iii) the colloidal gold immunochromatography assay (GICA); (iv) indirect hemagglutination assay (IHA); (v) DIGFA; (vi) the dipstick dye immunoassay (DDIA); (vii) polyacetal polystyrene immunization microspheres (PAPS); and (viii) circulating cathodic antigen (CCA).

Enzyme-linked immunosorbent assay (ELISA) There are a few ELISA-based serologic tests developed for detection of animal schistosome infection. Among them, the ELISA test that uses soluble egg antigen (SEA) as the target is the most widely used technique for a range of hosts, such as buffalo, goat, mice, rabbit, rodents, and sheep [73,74,77–79]. Some recombinant protein-based ELISA (e.g., SjTPx-1) tests were developed and tested in buffalo [74].

Indirect hemagglutination assay (IHA). IHA is a commonly used alternative test to detect human and animal infections with *Schistosoma* [79,84,87]. The test uses erythrocytes coated with schistosome adult worm antigen and has been widely used in diagnosis of *S*. *japonicum* infections in buffalo and cattle in the People’s Republic of China.

Circumoval precipitin test (COPT). The test is a simple and inexpensive immunodiagnostic test used to detect serum antibodies to schistosome and developed for diagnosis of schistosome in humans [88,89]. The test was adapted for detection of schistosome infection in animals such as buffaloes [74].

Dipstick dye immunoassay (DDIA). DDIA was developed for detection of antibodies against *S*. *japonicum* in humans using SEA labelled with a colloidal dye [90]. The technique was subsequently extended to diagnosis of animals [80].

The colloidal gold immunochromatography assay (GICA). GICA combines with the double-antigen sandwich assay and has been used for detection of antibody caused by schistosome infection. Most current applications focus on *S*. *japonicum* in a wide range of animal hosts [70,73,79].

The dot immunogold filtration assay (DIGFA). DIGFA is a rapid technique, based on an immune labelling technique developed in the late 1980s, for detection of antibodies to infectious agents. The technique was developed for detection of anti-*S*. *japonicum* antibody in humans in the People’s Republic of China [91] and then extended to diagnosis of *S*. *japonicum* infection in cattle [69,78].

Polyaldehyde polystyrene immunization microspheres (PAPS). This technique for schistosome detection was developed in late 1980s in the People’s Republic of China. It uses immunospheres of polyaldehyde polystyrene to link with special antigenic ligates (SEA of *S*. *japonicum*) and was used for diagnosis of *S*. *japonicum* in cattle in the People’s Republic of China [71,81].

Circulating cathodic antigen (CCA) assay. This technique detects the presence of schistosome CCA released from adult worms and is a widely used technique for all three species of *Schistosoma* of main public health concern. In this review, CCA assay was used (in comparative test) only in one study pertaining to *S*. *mansoni* [75].

Molecular techniques

PCR-based techniques. The techniques detect (e.g., qualitative) and quantify (e.g., both qualitative and quantitative) *Schistosoma-*specific DNA from samples (e.g., fecal or urine, depending on species). Extensive studies across different *Schistosoma* species claim very high sensitivities and specificities. Both qualitative (classic PCR) and quantitative PCR (qPCR) have been explored for *S*. *japonicum* and *S*. *mansoni* in animals and evaluated in both laboratory and field settings [45,66,72–77].

### Comparisons of results from different diagnostic techniques

The 19 studies included in the review used 14 diagnostic tests—13 studies used one or more parasitologic assays, 14 studies used one or more immunologic assays, and nine studies used either PCR, or qPCR, or both (Table 2). Given the availability of comparable information within and across studies, the following comparisons and analysis were conducted.

**Table 2:** Result of different diagnostic techniques used in the included studies (number of test positive/numbers of tested).

	Diagnosis	Buffalo	Cattle	Goat	Sheep	Dog	Mouse	Rabbit	Rodent	Chimp	Ref
**Parasitologic assay**	KK	0/10 11/44 11/106 3/81	8/10	37/145		0/52			12/30	1/19	[45] [50] [63] [66] [75] [76]
	MHT	1/10 4/19 7/8 0/81 9/197	10/10 5/162 139/139	40/145 16/314		0/52				1/9	[45] [50] [63] [66] [70] [69] [75]
	DBL	3/81									[66]
	FEA-SD	58/105 41/44	37/48								[72] [50]
**Immunoassay**	ELISA	49/178	140/140 109/110 94/94		106/107		50/50	30/30 51/96	20/20	29/31	[73] [77] [69] [79] [78] [82] [75] [76]
	GICA	17/197	14/162	32/314	98/107		50/50	30/30			[73] [70] [79]
	IHA	15/197	71/2,277 94/94	32/314				51/96			[70] [81] [82]
	T-DIGFA		279/279 110/110								[69] [78]
	PAPS		95/2,277								[81]
	CCA									10/20	[75]
**Molecular assay**	PCR	7/8 40/178									[63] [77]
	qPCR	9/10 34/66	10/10 42/48	10/145			9/49		15/20	13/24	[45] [72] [50] [66] [75] [76]

#### Parasitologic, immunodiagnostic, and molecular techniques

Two studies (both pertaining to *S*. *mansoni*) used all three types of diagnostic techniques. In the first study that examined intestinal schistosome infections in wild-born chimpanzees in Uganda, Standley et al. [75] reported results of different diagnostic tests with substantial variations in outcome measures; proportions of test positive were 5.3%, 93.5%, 50.0%, and 54.2% by duplicate KK thick smears, ELISA, CCA, and qPCR tests, respectively. The second study used the KK, ELISA, and PCR tests on experimental rodents, yielding results of proportions of test positive at 68%, 100%, and 79%, respectively [76].

#### Parasitologic and immunodiagnostic techniques

Five studies (all pertaining to *S*. *japonicum*) used the two types of diagnostic techniques. For parasitologic tests, all studies used MHT, and for immunologic tests, six of them (ELISA, DDIA, IHA, PAPS, DIGFA, and GIGA) were involved in the studies. Jiang et al. [68] used MHT and ELISA to examine cattle in an endemic area of Hunan province, People’s Republic of China and reported 13.4% and 18.5% of proportion of positive by the two tests, respectively. Sun and Zhang [71] compared MHT and PAPS tests in cattle in a highly endemic area of Hubei province, where both tests showed 100% prevalence of schistosome infections among cattle. Peng et al. [70] compared the use of MHT, GICA, and IHA in goat, buffalo, and cattle, showing significant variations in positive detected by MHT (in the range of 3.1% to 5.1%) and GICA/IHA (in the range of 7.4% to 10.2%). Lu et al. [69] examined the performance of MHT, ELISA, and DIGFA, all showing 100% proportion of positive in cattle in a field study in Zhejiang province, People’s Republic of China.

#### Parasitologic and molecular techniques

Eight studies used molecular (PCR and/or qPCR) and parasitologic (KK, MHT, DBL, and FEA-SD) techniques (Table 2). In the study on buffalo in the Philippines, Wu et al. [66] compared qPCR vs. KK, MHT, and DBL, and found substantial variations in schistosome detection associated with the different tests–the three parasitologic assays reported proportions of test positive from 0 to 3.7%, while qPCR test indicated about 51.5% proportion of positive tests. In another study pertaining to buffalo also conducted in the Philippines, Gordon et al. [72] evaluated the performance of KK, MHT, a newly developed parasitologic test, FEA-SD, and qPCR, showing that 25.0% and 19.1% proportions of test positive were identified by KK and MHT, while the FEA-SD and qPCR picked up 93.2% and 90.9% of the test positives, respectively. Fung et al. [63] compared MHT and PCR in the detection of schistosome infection in bovine, which gave the same result (62.5% test positive). There were two studies on *S*. *mansoni* conducted in Brazil and Uganda, respectively. The Brazilian study used KK and PCR tests on rodents and found 65% (KK) and 75% (PCR) proportions of test positive, respectively [76], while the study on chimpanzee in Uganda reported test positive at 5.3% by KK, 1.1% by MHT, 93.5% by ELISA, 50.0% by PCR, and 54.2% by qPCR [75].

**Fig 2:**
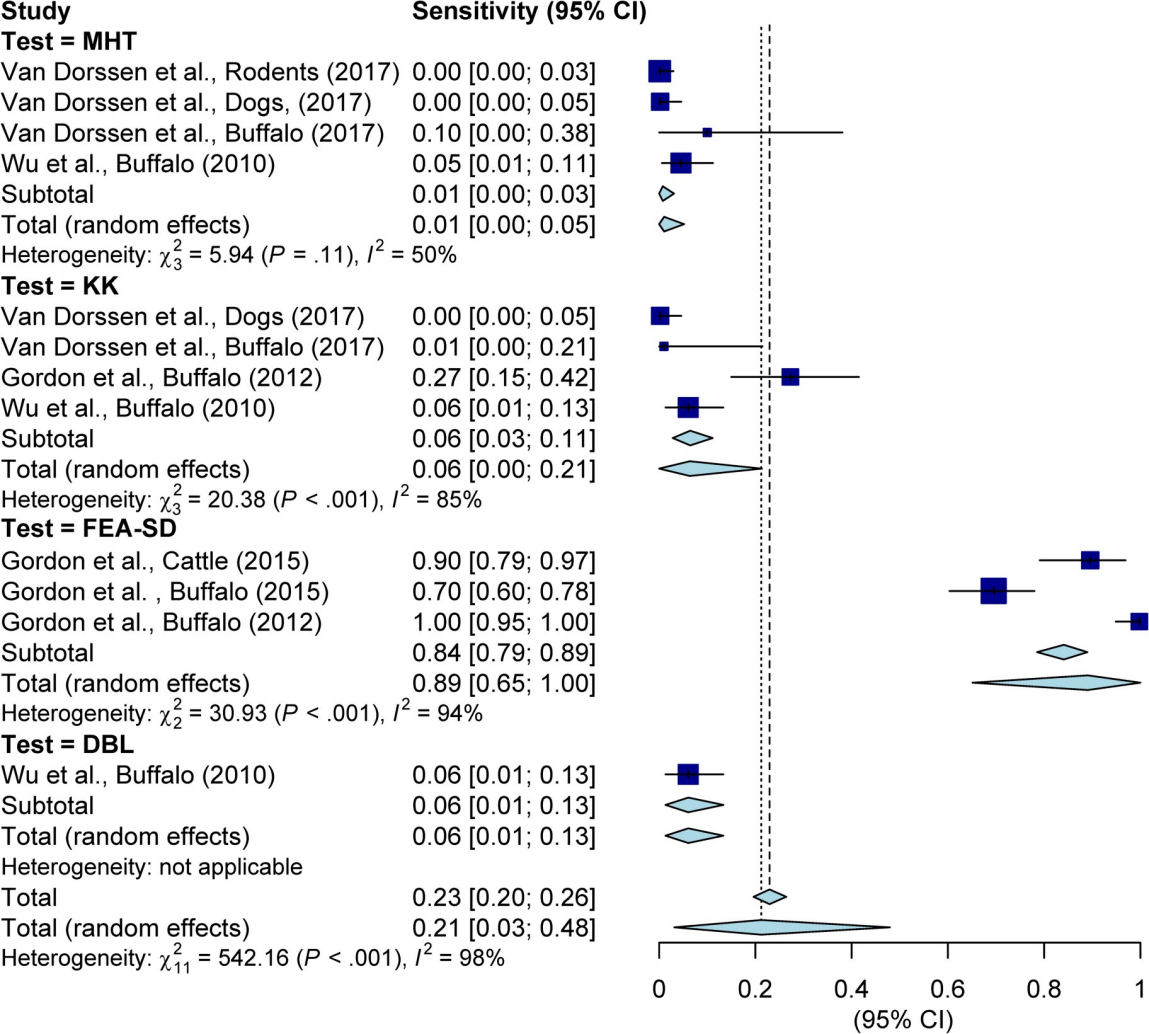
Meta-analysis of sensitivity of selected diagnostic techniques for the diagnosis of schistosome in non-human animals.

Given the availability of comparable data, four studies were included in meta-analysis of sensitivity analysis of the four parasitologic techniques, using qPCR a reference test. The four studies, all pertaining to *S*. *japonicum*, used one or two of parasitological tests (KK, MHT, DBL, or FEA-SD) and qPCR. Estimates of sensitivity associated with each of the four parasitologic assays were included in the meta-analysis, which showed substantial variations over parasitologic tests across different hosts. The pooled estimates of sensitivity for MHT, KK, FEA-SD, and DBL tests were 0.01 (95% CI: 0–0.05), 0.06 (95% CI: 0–0.21), 0.89 (95% CI: 0.65–1.0), and 0.06 (95% CI: 0.02–0.15), respectively, with the overall estimate of 0.21 (95% CI: 0.03–0.48) (Fig 2).

## Discussion

Over the past 30–40 years, several diagnostic techniques have been developed and adapted for the detection of *Schistosoma* infection in non-human hosts. Some of these techniques have been widely used, while others have had limited application in practice. Establishing optimal techniques for field application is challenging, as the ‘effectiveness’ of a diagnostic test depends on a wide array of factors, such as parasite biology, transmission levels of the parasite, species of animal hosts involved, and local infrastructure and capacity, among others. Here, by means of a systematic review, 19 studies were identified that employed 14 diagnostic techniques, classified as parasitologic, immunologic, or molecular (Table 1). Consistent, quantitative comparisons could not be undertaken for many of the reported techniques for lack of comparable published data, particularly for immunodiagnostic assays. Quantitative comparisons were performed between four parasitologic assays and qPCR for a subset of studies. While limited, these comparisons yielded valuable information and identified important, remaining knowledge gaps.

As for diagnosis of human schistosome infections, parasitologic tests, in particular direct microscopic techniques such as KK and MHT, are widely used for the diagnosis of zoonotic schistosome infections. Important limitations of these techniques are widely recognized. First, their test sensitivities decrease with the declining infection intensity or level of transmission [13, 23,26,35]. Second, for diagnosis of schistosome infection in animals, in particular large animals such as buffalo and cattle, which play an important role in schistosomiasis transmission to humans in parts of East Asia, the MHT was preferred over the KK due to the use of much larger size of fecal samples, yielding a better performance of diagnosis based on extensive fieldwork reported in the People’s Republic of China [32,35,63,86]. However, it should be noted that MHT results were highly sensitive to pH, temperature, and quality of the water used in the hatching assays and if not controlled well, could significantly impact test results [86]. A recent study in West Africa on the detection of *S*. *bovis* and *S*. *curassoni*, as well as hybrids in livestock (cattle, goat, and sheep) using KK and MHT techniques (and using abattoir data to estimate sensitivities of the tests used) also suggested that overall MHT tests had significantly higher sensitivities than KK for cattle, although there were significant differences in the performance of the MHT depending on infecting schistosome species [8]. In contrast, there were no significant differences in estimates of test sensitivity by parasite species in sheep and goats, nor between proportions testing positive by each test [8].

Among the four parasitologic techniques, the FEA-SD, a recently developed microscopic test, has shown high sensitivity and specificity [86]. The procedure involved in the FEA-SD technique is relatively straightforward and easy to implement under field conditions. This technique is an important improvement, overcoming the limitations of classic parasitologic techniques in detecting schistosome eggs in animals–for example, the feces of large animals (e.g., bovine) typically comprise large masses containing cellulosic fiber and abundant debris that obscure egg detection under a microscope using the classic KK test. The FEA-SD procedure can clear large proportions of the debris, enabling more efficient observation [86]. Using the qPCR as the reference test, the sensitivity of each of the four tests was estimated for the four eligible studies, and meta-analysis shows that the FEA-SD has the highest pooled estimate of sensitivity, 0.89 (95% CI: 0.61–1.00) in contrast with much lower estimates for the other three parasitologic assays (Fig 2). A recent study in the Philippines examined performance of FEA-SD and qPCR in the diagnosis of *S*. *japonicum* infection among buffaloes post-natural exposures at different time points, suggesting an overall sensitivity of 75.8% by FEA-SD, and 87.9% by qPCR, up to 93.9% for combination of both, using buffalo perfusion results as the reference [92]. The FEA-SD provides an overall similar level of diagnostic accuracy as qPCR. Given the much lower cost of diagnosis using FEA-SD compared to that of qPCR, the newly developed parasitologic technique has the potential as an affordable test for detection of schistosome infection in animals. It should be noted that, in the study by Van Dorssen and colleagues [45], the performance of both KK and MHT in the diagnosis of goats was better than that of qPCR, resulting in high sensitivity of both tests using qPCR as a reference. The low performance of qPCR in comparison with KK and MHT was unexpected and the authors later identified that this might be due to presence of inhibitors in the goat stools [45]. Hence, the information on goats was not included in the meta-analysis. It should also be noted that, although not included in the review due to lack of comparisons, a non-invasive technique, mini-FLOTAC, was used for detection of schistosome and other trematode infections in wildlife with promising results [93].

In addition to the comparisons included in the present study, we identified studies reporting on promising results of immunodiagnostic tests. A recent study examining thioredoxin peroxidase-1 in an ELISA system showed effective identification of *S*. *japonicum* in bovine hosts [74]. There are some other widely applied immunodiagnostic techniques in the field (e.g., indirect immunodiagnostic assays detecting specific schistosome induced antibody including COPT and DDIA), which demonstrate high sensitivity but generally low specificity [26]. Circulating antigen based detection has a relatively high sensitivity, whereas the specificity becomes an issue, particularly in low transmission settings [26]. Nevertheless, it is widely recognized that immunodiagnostic techniques face many challenges, in particular those related to cross-reactivity and identification of past infections, rather than current infections. These issues have limited, to some extent, their value as effective tools for detection of schistosome infections.

Note that only two studies pertaining to *S*. *mansoni* involved comparative diagnostic tests, and hence, were included in the review. The Gentile et al. [76] study compared KK, ELISA, and PCR in 20 experimental rodents and found that ELISA generated highest detection rate (100%), followed by PCR (75%) and KK (65%). Standley et al. [75] pursued a cross-sectional survey on chimpanzees in Uganda using KK, MHT, ELISA, CCA, and qPCR and reported highest detection by ELISA (93.5%), qPCR (54.2%), CCA (50.0%), KK (5.3%), and MHT (1.1%). However, no specific comparison across studies and tests could be made due to lack of cross-tabulated information. The overall patterns of findings are in general agreement with those on *S*. *japonicum*. It is worth noting that, using molecular techniques (e.g., ITS/COX-1 and PCR), some recent studies have offered important data suggesting non-human primates [94] and rodents [56] as reservoirs for *S*. *mansoni*. Although future research is needed to deepen the understanding of these and other potential reservoirs to human infection, these studies offer important and promising prospect for diagnosis of animal schistosome, particularly in the context of elimination of human *Schistosoma* transmission. Highly sensitive and accurate techniques will be the key to verification of transmission interruption.

This study is subject to a couple of limitations. We recognize that the number of studies included in the analysis is small, and sample sizes of the included studies varied substantially–some having small sample sizes–which may cause bias in the pooled estimate of meta-analysis. In addition, it should be noted that lack of information on appropriate quality control measures for diagnostic techniques (particularly molecular techniques) poses challenges to assessment of diagnostic tools, and participation in an external assessment schedule, such as the one already initiated for human molecular diagnosis [95], should be encouraged also for the diagnosis of schistosome infection in non-human animal hosts.

Taking together, having reviewed and analyzed available data, we found that diagnostic techniques across the three categories exhibit substantial heterogeneities in their strengths and limitations with respect to *Schistosoma* diagnosis in non-human animals. The FEA-SD parasitologic technique, and molecular techniques, especially qPCR, are potentially promising, and field-applicable techniques for schistosome diagnosis in non-human animal hosts. Future studies are needed for validation and standardization for their broader applications under real-world conditions.
